# How (Epi)Genetic Regulation of the LIM-Domain Protein FHL2 Impacts Multifactorial Disease

**DOI:** 10.3390/cells10102611

**Published:** 2021-10-01

**Authors:** Jayron J. Habibe, Maria P. Clemente-Olivo, Carlie J. de Vries

**Affiliations:** 1 Department of Medical Biochemistry, Amsterdam University Medical Centers, Amsterdam Cardiovascular Sciences, and Amsterdam Gastroenterology, Endocrinology and Metabolism, 1105 AZ Amsterdam, The Netherlands; j.j.habibe@amsterdamumc.nl (J.J.H.); m.p.clemente@amsterdamumc.nl (M.P.C.-O.); 2 Department of Physiology, Amsterdam University Medical Centers, Amsterdam Cardiovascular Sciences, 1081 HV Amsterdam, The Netherlands

**Keywords:** obesity and related metabolic diseases, epigenetic marks, transcription factors, metabolic diseases, gene expression

## Abstract

Susceptibility to complex pathological conditions such as obesity, type 2 diabetes and cardiovascular disease is highly variable among individuals and arises from specific changes in gene expression in combination with external factors. The regulation of gene expression is determined by genetic variation (SNPs) and epigenetic marks that are influenced by environmental factors. Aging is a major risk factor for many multifactorial diseases and is increasingly associated with changes in DNA methylation, leading to differences in gene expression. Four and a half LIM domains 2 (FHL2) is a key regulator of intracellular signal transduction pathways and the *FHL2* gene is consistently found as one of the top hyper-methylated genes upon aging. Remarkably, *FHL2* expression increases with methylation. This was demonstrated in relevant metabolic tissues: white adipose tissue, pancreatic β-cells, and skeletal muscle. In this review, we provide an overview of the current knowledge on regulation of *FHL2* by genetic variation and epigenetic DNA modification, and the potential consequences for age-related complex multifactorial diseases.

## 1. Introduction

**Figure 1:**
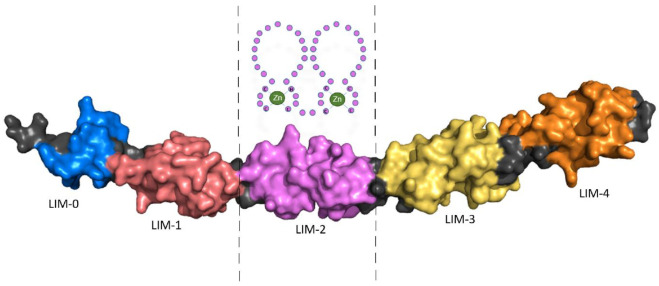
Schematic representation of LIM domain 2 (LIM-2) composed of two zinc fingers, above, with the complete protein structure of FHL2 based on X-ray structures of the independent LIM domains and Alphascreen computation (AF-Q14192-F1-model_v1.PDB: https://alphafold.ebi.ac.uk/). Accessed in July 2021.

Four and a half LIM domains 2 (FHL2) was originally described as ‘Down-regulated in Rhabdomyosarcoma LIM protein’ (DRAL) and is composed of LIM domains that are named after their initial discovery in the proteins Lin11, Isl-1 and Mec-3 [1]. FHL2 consists of four and a half LIM domains, each composed of two zinc fingers, except the first ‘half’ LIM-domain which has only one. While the structure of the four and a half LIM domains had been uncovered previously using Nuclear Magnetic Resonance (NMR) spectroscopy, the complete FHL2 protein structure is unknown; however, it has recently been predicted using the protein structure neural network AlphaFold (Figure 1) [2].

The LIM domains allow FHL2 to interact with a variety of other proteins, resulting in an impressive interactome comprising more than 92 targets known to date [3]. Upon binding of FHL2, the conformation of target proteins may alter and consequently their post-translational modifications, their cellular localization, or their interactions with other proteins may change. Thus, FHL2 acts as a scaffold protein and can adjust the structure, activity, and function of its interaction partners. In contrast to the findings regarding many zinc fingers which indicate their ability to bind to DNA, the zinc-fingers of FHL2 lack this ability. Therefore, FHL2 does not directly regulate gene expression. However, FHL2 is of course still able to regulate gene expression via interaction with transcription factors and their upstream co-regulators [4]. Through binding to its target proteins, FHL2 modulates and fine-tunes signal transduction pathways and subsequent gene regulation, which is important in the function of various tissues and their pathologies.

*FHL2* is expressed most abundantly in the heart, blood vessels, ovary, and skeletal muscle, and to a lesser extent in several other organs [5,6,7,8]. FHL2 expression and function have been studied in a number of diseases, including various types of cancer [9,10,11,12], cardiovascular disease [5,6,8,13,14,15], and overall metabolism [16]. At present, information regarding the exact regulation of *FHL2* expression is limited [17,18]. Several genome-wide association studies (GWAS) have shown association between *FHL2* single nucleotide polymorphisms (SNPs) and complex diseases and traits [19,20,21]. While informative, *FHL2* SNPs alone are unlikely to be sufficient to elucidate the role of FHL2 in complex multifactorial traits and maladies.

Growing evidence supports the notion that epigenetics, and in particular DNA methylation, mediates the connection between environment and gene expression across the human body. The human DNA methylation pattern or ‘methylome’ shows considerable variation and is indeed responsive to environmental cues [22]. Actually, several risk factors for disease such as age, unhealthy diet or exposure to chemical agents play a decisive role in the methylation status of the genome [23]. The *FHL2* gene contains a number of methylation-prone CpG sites that have repeatedly been shown to become methylated with age and which are involved in the regulation of *FHL2* expression.

These methylated *FHL2* CpG sites have also been shown to be quite consistently present in different tissues, while demonstrating significant correlations with aging [24,25,26]. These recent findings suggest a potentially pivotal role for methylation in regulating FHL2 expression and function in age-related disorders.

## 2. *FHL2* Tissue Expression

**Figure 2:**
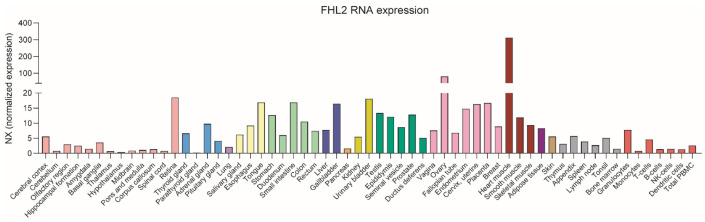
Normalized FHL2 mRNA expression in multiple tissues. Based on transcriptomic data from three sources: HPA, GTEx, and FANTOM5 (Protein Atlas: https://www.proteinatlas.org/). Accessed in July 2021.

*FHL2* was initially discovered as one of the genes strongly down-regulated during transformation of healthy primary myoblasts into rhabdomyosarcoma tumor cells [27]. Next, it was shown that *FHL2* is expressed in multiple tissues, with the highest expression level found in the heart [28]. However, substantial levels of expression are also present in the ovaries, testes, prostate, and intestine (Figure 2). The relatively high *FHL2* expression in the heart explains why its function has been the subject of extensive study in this organ. In early embryonic development FHL2 is already present in the heart, and it remains there into adulthood in both mice and humans. FHL2 is dispensable for normal development of the heart in mice, as illustrated by the observation that the heart matures normally in *FHL2*-deficient mice [13]. In adult mice, *FHL2* deficiency causes an increased hypertrophic response in cardiac disease models such as β-adrenergic stimulation to provoke cardiac hypertrophy or the coronary artery ligation model to induce ischemia [28,29].

In skeletal muscle, FHL2 promotes differentiation of myoblasts and is involved in autophagy [7,30]. It is expressed in almost all areas of the developing brain but restricted to particular regions postnatally, mainly the cortex [31,32]. It was demonstrated that *FHL2* deficiency affects neuroblast migration, resulting in accumulation of specific astrocytes in the brain of mice. Furthermore, FHL2 plays a role in ocular vascularization [33]. Based on experiments in *FHL2*-knockout (KO) mice it was concluded that FHL2 promotes corneal angiogenesis by inhibiting local inflammation. In the liver *FHL2* levels are relatively low, but upon overexpression in this organ in mice the balance of hepatocyte proliferation and apoptosis is disturbed, leading to inflammation and cirrhosis. Furthermore, in *FHL2*-KO mice the liver shows increased fibrosis and an exacerbated response in a cholestatic injury model [34,35,36]. FHL2 has been detected in kidney podocytes where it interacts with and activates the Wnt/β-catenin signaling pathway [37]. Intriguingly, though *FHL2* is not highly expressed in either spleen or immune cells, it is found to regulate inflammation in stressed or injured tissues [38,39]. Atherosclerosis, which may be considered a chronic inflammation of the vessel wall, was studied in *FHL2*-KO mice; these developed smaller atherosclerotic plaques after a cholesterol-enriched diet. This can be at least partly explained by decreased chemokine production resulting in reduced monocyte recruitment to the vessel wall [40,41]. Conflicting data have been reported on the function of FHL2 in vascular smooth muscle cells in regulation of proliferation, inflammation, and cholesterol efflux of these cells, which has been reviewed before [4,5,8,42]. 

Bacos et al. revealed that age-dependent DNA methylation of the *FHL2* gene correlates with increased *FHL2* expression in human pancreatic islets [38]; this will be described in more detail in the paragraph on *FHL2* methylation below. A similar pattern was observed in adipose tissue, with enhanced DNA methylation of the *FHL2* gene resulting in higher expression levels [43]. *FHL2* expression in adipose tissue correlates with increased body mass, and it has recently been demonstrated that *FHL2*-KO mice are resistant to weight gain involving increased ‘browning’ of white adipose tissue [16].

In a number of cancer cells, FHL2 protein has been described to function either as a tumor suppressor or as an oncoprotein. Basal and stimulated expression of *FHL2* appears to be highly influenced by the tumor cell type, tumor origin, and context [44,45]. Furthermore, FHL2 plays distinct roles in breast, ovarian, and prostate cancer through its interaction and regulation of transcription factors such as the estrogen and androgen receptor [12,46,47,48,49]. *FHL2* expression and function also varies in cancer of the liver, tongue and gastrointestinal tract [50,51,52,53]. The complicated role of FHL2 in cancer will not be described in further detail in this review.

## 3. Regulation of *FHL2* Expression by Specific Transcription Factors

There is only limited information concerning the regulation of *FHL2* gene expression by specific transcription factors. Using chromatin-immunoprecipitation (ChIP), the tumor suppressor protein p53 has been demonstrated to induce *FHL2* expression through selective binding of a response element localized in one of the two alternative promoters of *FHL2* [50]. Specificity protein 1 (SP1), an essential transcription factor in many cancer cells, has also been shown to regulate *FHL2* expression by binding upstream of the FHL2 transcription start site [54]. In hepatocellular carcinoma cells, expression of the proteins Paired Box 5 (PAX5) and Zinc Finger Protein 5 Homolog (ZFP5) positively correlate with FHL2 expression, while potential binding sites for these proteins were found in the *FHL2* promoter sequence [17]. Similarly, Myocyte Enhancer Factor 2 (MEF2), a transcription factor relevant in cardiac and skeletal muscle, has a potential binding site in the *FHL2* promoter and shows expression overlapping with *FHL2* in these tissues [32]. Furthermore, *FHL2* is a target gene of Serum Response Factor (SRF), which together with its interaction partner, the Homeobox Protein Nkx2.5, regulates *FHL2* expression [32,55]. In osteoclasts, FHL2 forms a complex with RUNX Family Transcription Factor 1 (RUNX1) and TNF Receptor Associated Factor 6 (TRAF6) to control its own expression via RUNX1 binding sites [18]. Another example of autoregulation involves Activator Protein 1 (AP1), a known FHL2 interactor, which binds and promotes expression of the *FHL2* gene [56].

## 4. FHL2 Genetic Variation 

**Figure 3:**
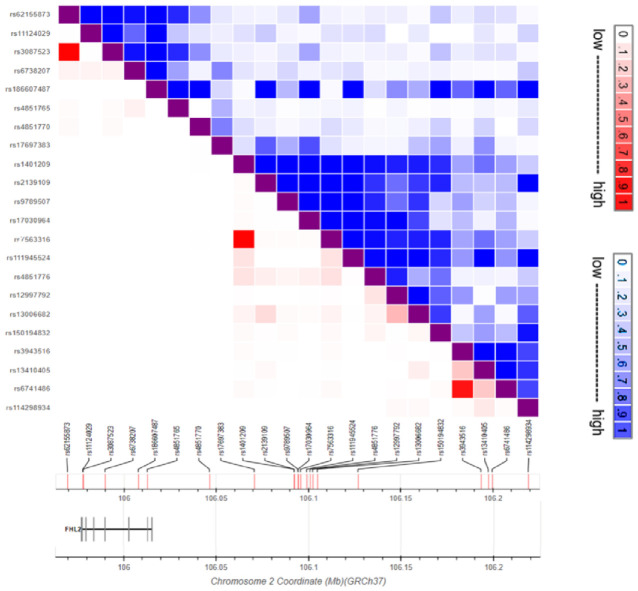
Schematic representation of SNPs in an LD matrix and their localization on chromosome 2, with the *FHL2* gene indicated at the left (GrCh37). SNPs with high LDs are indicated in red (R^2^) and blue (D’). Figure adapted from LDlink.

At present, 22 *FHL2* SNPs have been associated with a specific disease or trait: cardiac myopathy, pulse pressure, venous thrombosis, severity of bronchial hyper-responsiveness, acute myeloid leukemia, myeloid white cell count, platelet count, fat body mass, BMI, age of menopause, the electrocardiographic PR-interval of the heart, lung cancer in ‘ever-smokers’, smoking status, height, and unipolar depression. These data are summarized in Figure 3 and Table 1 and we will describe current knowledge about these *FHL2* genetic variants in more detail. The two SNPs that are located in the coding region of the *FHL2* gene are both synonymous variants not giving rise to amino-acid sequence changes. Furthermore, for the variants that are located in non-coding regions of the *FHL2* gene, we envisage that they are either neutral, lead to alternative splicing of the *FHL2* gene, and/or influence *FHL2* expression.

### 4.1. FHL2 SNPs and Metabolic Phenotypes

There is an emerging focus on FHL2 function in the context of metabolism [16,68], which is also reflected by the observation that rs3087523 and rs186607487 are associated with body mass index (BMI) and fat body mass, respectively (Table 1) [60,61,62]. In line with this observation, *FHL2*-deficient mice gain less weight than their wild-type littermates in response to a high-fat diet [16]. In the latter study, ‘browning’ of white adipocytes was observed in the mice upon *FHL2* deficiency. Similar changes in gene expression were observed in human white adipose tissue. In humans, increased *FHL2* expression is also associated with decreased expression of a number of the so-called ‘browning genes’, such as peroxisome proliferator-activated receptor gamma coactivator 1-alpha (*PGC1a*), cardiolipin synthase 1 (*CRLS1*), sirtuin 1 (*SIRT1*), peroxisome proliferator-activated receptor gamma (*PPARG*), and bone-morphogenic protein 7 (*BMP7*) [16]. Of note, the variants rs3087523 and rs62155873 are in high linkage disequilibrium (LD) and are both associated with BMI in the same study. In another study, rs3087523 was shown to associate with BMI (Figure 3) [61]. Kichaev et al. *FHL2* revealed that the genetic polymorphism rs6738207 is associated with height. While this information is only present within the supplementary data, it is still of note as height is a major determinant of BMI and may be of interest to investigate further.

### 4.2. FHL2 SNPs in Cardiovascular Disease and Lung Inflammation

FHL2 has been studied extensively to determine its role in both the heart and the vasculature, as described above. Two *FHL2* polymorphisms, rs3087523 and rs11124029, have been linked to hypertrophic cardiac myopathy [63]. So far, it is unknown for both of these variants which of their respective alleles is actually the risk allele associated with developing cardiac myopathy. Additionally, *FHL2* polymorphisms rs13006682 and rs150194832 have been linked to the PR interval of the heart and pulse pressure, respectively [64,67].

The *FHL2* genetic variant rs4851765 associates with the severity of bronchial hyper-responsiveness [57]. Here, the risk allele is also unknown, as rs4851765 has two alternative alleles. In line with this observation in humans, Kurakula et al. demonstrated that mice lacking *FHL2* have a weakened airway inflammatory response in an allergenic respiratory inflammation mouse model.

### 4.3. FHL2 SNPs in Coagulation and Cancer

Kroone et al. demonstrated in mice that *FHL2* deficiency enhances venous thrombosis. FHL2 interacts with Tissue Factor resulting in a reduction of its coagulation activity, and there is thus enhanced blood clot formation in *FHL2*-deficient mice. Furthermore, *FHL2* SNP rs4851770 was shown to associate with the risk of developing venous thrombosis in humans [59]. Given the specific location of this SNP, it has been classified as an upstream transcript variant that forms part of intron 1 in two *FHL2* transcript variants, but not in others. To date, two studies have shown an association between *FHL2* SNPs and blood platelet count [65,66]. The relevant SNP variants are rs3943516 and rs6741486; both are located in the intergenic region upstream of the *FHL2* gene (Table 1). These same variants are in LD and have additionally been associated with myelopoiesis and hematopoiesis [69]. As indicated above, FHL2 is often encountered in the context of oncology, and a large body of research implicates FHL2 in the development of leukemia [69,70,71,72,73]. Several of these studies showed that increased *FHL2* expression correlates with poor prognosis of leukemia [69,70]. One GWAS has shown an association between *FHL2* variants rs1401209, rs9789507, rs7563316, rs17030964, rs4851776, rs2139109, and rs12997792 and acute myeloid leukemia (Table 1) [19]. Of note, all these genetic loci are intergenic variants located outside of the *FHL2* gene. McKay et al. revealed the association between *FHL2* SNP rs17697383 and lung cancer in smokers [20]. While it was not their primary finding, it is still important to note that this SNP is associated with ‘ever-smoking’, with the major C-allele serving as the risk allele. *FHL2* polymorphism rs62155873 is associated with smoking as well. 

### 4.4. FHL2 SNPs and Diverse Traits

Howard et al. performed a GWAS to identify genetic loci associated with depression-related phenotypes. As can be garnered from the supplementary data, *FHL2* genetic locus rs111945524 is associated with broad depression [21]. *FHL2* SNP rs114298934 was reported as an SNP that associates with age of menopause in a population enriched for longevity [58]. In this specific study, it was also reported that this genetic variation does not impact *FHL2* gene expression. 

Taken together, the 22 *FHL2* genetic polymorphisms listed here show a range of associations with various metabolic, cardiovascular, and oncological diseases and traits, which are predominantly age-related conditions. While the role of the *FHL2* SNPs in these fields is uncertain, given that they are either synonymous variants or located within the non-coding/ intergenic regions we can still conclude that the contribution of genetic variation in the *FHL2* gene on its own in numerous phenotypes seems limited. 

## 5. Methylation of the *FHL2* Gene

DNA methylation is the best-known epigenetic modification of the genome, altering DNA accessibility and thereby regulating gene expression. It occurs when a methyl group is added enzymatically to a cytosine, usually forming part of a CG dinucleotide (CpG). Genomic regions where CpG dinucleotides or sites cluster are called CpG islands. They are usually located in the proximity of a promoter region. However, methylation of CpG sites can also occur in other areas such as exons, introns or intergenic regions [74]. Patterns of DNA methylation are constantly changing from early stages of development through physiological processes and pathological conditions, strongly influenced by the environment [23]. Actually, several environmental factors such as age, diet, physical activity, microbiota or exposure to chemical and physical agents play a major role in disease progression through the DNA methylation changes they produce in the genome [23,75]. In the next sections, we will summarize the current literature on DNA methylation of *FHL2* and its relevance in aging and age-related diseases, including obesity and type 2 diabetes.

### 5.1. Hyper-Methylation of FHL2 in Aging

**Figure 4:**
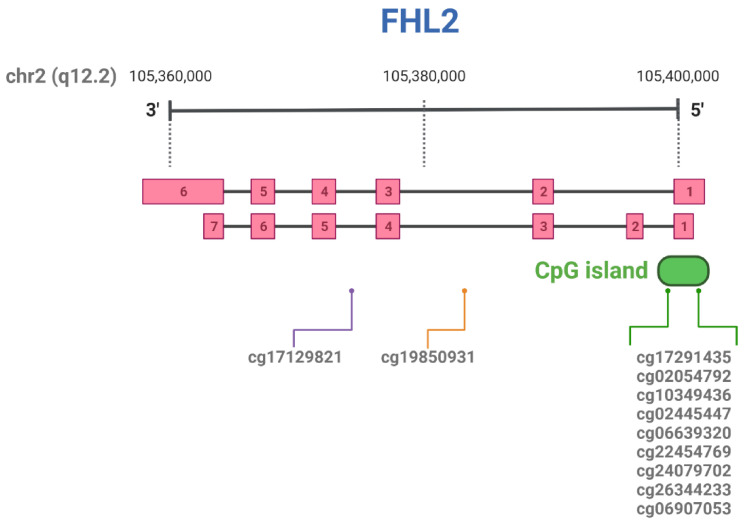
Schematic representation of *FHL2* transcript variants (exons are sequentially numbered) and relevant CpG sites, according to Genome Browser (GRCh38/hg38 and GRCh37/hg19). Created with BioRender.com.

There is an “epigenetic clock”, which consists of specific methylation changes located in particular loci of the human genome that can be found across multiple tissues, which individuals of the same age have in common [76]. Age-prediction is particularly useful in forensic science, where determination of the age of an individual in limited amounts of DNA is key. In a fascinating turn of events, a person’s age can be assessed by determining the extent of methylation of specific CpG sites in the genome. In 2013, Hannum et al. developed a mathematical model for age prediction based on methylation of 71 CpG sites in different genes, and since then efforts have been made to simplify and refine this biological age prediction tool [77]. One of the hyper-methylated loci most consistently found upon aging is located in the *FHL2* gene, together with loci in Elongation of Very Long Chain Fatty Acids Protein 2 (*ELOVL2*), Kruppel-like factor 14 (*KLF14*) and Proenkephalin (*PENK*) [43,78,79,80]. More specifically, there is a region of clustered CpG sites (a CpG island) located proximal to the first exon of the *FHL2* gene (Figure 4). The majority of *FHL2* CpG sites found hyper-methylated with aging in multiple tissues are localized in this region of chromosome 2. The relevant CpG sites for *FHL2* that have been described in more detail are summarized in Table 2 and Figure 4. Three methylation sites in the CpG island of *FHL2* are included in the methylome-based age-prediction model: cg06639320, cg22454769 and cg24079702 [77]. A study comparing DNA methylation in blood from mother-offspring couples revealed these three sites within the top five most significantly methylated CpG sites. This result was further confirmed in larger cohorts including males and females from a wide age range (9–99 years old) or middle-aged individuals [26,78,81]. Recently, forensic studies have expanded the use of the age-prediction tool to other samples apart from blood, such as saliva, bone or buccal swabs, and also confirmed its use in living and deceased individuals. In all of the samples except buccal swabs, at least one of the aforementioned three CpG sites for *FHL2* is consistently used as part of the array of CpG sites used for age determination [79,82,83,84,85,86,87]. Even though a study performed in bone alone did not find a significant correlation of age to *FHL2* methylation sites [25], in teeth, the methylation of 8 CpG sites in *FHL2*, together with sites in *ELOVL2* and *PENK*, can accurately predict age [88]. An additional tissue where *FHL2* methylation on cg22454769 was found to correlate with age is skeletal muscle [89]. Some pathologies are closely associated with accelerated cell aging and deterioration, such as Alzheimer’s Disease and Grave’s Disease, distorting the link between biological and chronological age. To evaluate the age prediction accuracy of reported methylation-based age-prediction markers, among which is *FHL2*, the methylation pattern of individuals who suffer from these two diseases was analyzed in blood. Patients with Alzheimer’s Disease showed normal *FHL2* methylation, whereas in the blood of patients with Grave’s Disease a marked hypomethylation was found for one of the *FHL2* CpG sites [90]. Exposure to certain chemicals is known to affect DNA methylation and is also associated with disease development. Among the toxic compounds to which many individuals are continuously exposed are polycyclic aromatic hydrocarbons (PAHs), which increase the risk of cancer. Li et al. hypothesized that long-term exposure to PAH accelerates aging through increased methylation, which was confirmed by correlation of PAH presence in urine samples and hyper-methylation of *FHL2* and *ELOVL2* in an individual’s leukocytes. Interestingly, in this study increased methylation of FHL2 at cg22454769 was in line with increased expression [91]. Finally, a very extensive study performed in several tissues and cell lines aimed to evaluate the capacity of *FHL2* and *ELOVL2* to predict age. In order to do this, some previously reported CpG sites from both genes were used as probes (for *FHL2* these were cg06639320, cg22454769, cg24079702, cg26344233, and cg06907053), and samples from individuals with a wide range of age were tested. Though a consistent linear trend of hyper-methylation could be seen for *FHL2* across the majority of tissues, in blood only site cg06639320 significantly correlated to age [92].

### 5.2. Hyper-Methylation of FHL2 in Metabolism

BMI, Hba1c (a marker for diabetes), and epigenetic changes have been evaluated in human white adipose tissue and compared to the methylome in blood from the same individuals. Four CpG sites of *FHL2* were found to be associated with age in both blood and subcutaneous white adipose tissue in a male cohort; this concerns cg06907053, cg26344233 and cg10349436, located within the CpG island, and cg19850931, located in an intronic region. An additional site (cg22454769) was found hyper-methylated in the female validation cohort only [43]. It is important to stress again that hyper-methylation of the *FHL2* gene correlates with increased *FHL2* expression, which is rather uncommon as, in general, hyper-methylation of genes causes gene-silencing. In white adipose tissue, *FHL2* expression increases with methylation, as already mentioned above, which was also observed in blood and pancreatic islets of healthy and type 2 diabetic subjects [68]. More specifically, in the latter study hyper-methylation of cg06639320 and cg22454769 CpG sites was shown to correlate positively with age and *FHL2* expression. The same group studied methylation in the liver and compared this to blood methylomes of individual donors. This resulted in a list of 16 CpG sites, in which *FHL2* was not included. Yet, eight different CpG sites in *FHL2* were found significantly more methylated in the aged liver; the aforementioned cg06639320, cg22454769 and cg24079702, as well as cg06907053, one of the sites also found in white adipose tissue, and four additional CpG sites: cg02054792, cg02445447, cg17291435, cg17129821 [93].

Taken together, and considering the increasing number of studies based on the epigenetic clock, we can conclude unequivocally that methylation of the *FHL2* gene is associated with aging and is an important marker in age-related prediction tools. Furthermore, there is the highly interesting situation that hyper-methylation of CpG sites in the promoter region of *FHL2* seems to provoke increased gene expression. Even though in many cases DNA methylation of a promoter region inhibits the binding of transcription factors, thus silencing gene expression, this turns out to be less of a universal mechanism than previously thought. Advances in sequencing techniques have indeed uncovered that a large proportion of hyper-methylated regions correlate positively with gene expression [75,96]. Most likely, though still unproven, methylation of the *FHL2* promoter region interferes with binding of inhibitory DNA-binding factors, thereby facilitating gene expression.

## 6. Concluding Remarks and Perspective

An impressive number of studies have been performed to unravel the function of the protein–protein interactor FHL2 in several pathologies, ranging from cardiac dysfunction and vascular disease to cancer and obesity. In addition, as documented here, epigenetic and, to a lesser extent, genetic variation in *FHL2* correlates with specific diseases and traits. *FHL2*-deficient mice have been instrumental in assessing the functional involvement of FHL2 in these (patho)physiological processes.

Taking the data on (epi)genetic modification and function of FHL2 together, it is indisputable that hyper-methylation at specific CpG sites in the promoter region of the *FHL2* gene is associated with human aging. Unexpectedly, increased methylation of *FHL2* coincides with enhanced expression of this protein. So far, no data are available on whether, in addition to this epigenetic regulation of *FHL2* gene expression, genetic variation also modifies *FHL2* splicing and/or expression. Furthermore, we conclude that, in mice, FHL2 has a beneficial function in cardiac hypertrophy, skin and intestinal wound healing, corneal angiogenesis and cholestatic injury of the liver. This comes at a price, however, as high *FHL2* expression worsens the outcome of atherosclerosis and acute myeloid leukemia, as well as obesity and white adipose tissue function. Especially for atherosclerosis, cancer and obesity, aging is a well-known and severe risk factor. While the function of FHL2 is multi-faceted and ambiguous, these data collectively point towards a central and perhaps even fundamental role for FHL2 in the complex mechanism of aging and in its associated illnesses. In conclusion, detailed knowledge of the human methylome may lead to a better understanding of age-related molecular pathways that predispose individuals to obesity and its comorbidities. To assess whether FHL2 is indeed actively involved in aging, we need to unravel its manner of action in this complicated process in greater detail using dedicated mouse models, human databases and primary cell lines.

## Figures and Tables

**Table 1:** *FHL2* SNPs and associated traits. The table denotes the *FHL2* genetic polymorphisms that are associated with a disease or trait, according to the literature. Here, we indicate the reference allele (Ref), the alternative allele (Alt), the location of the SNP on chromosome 2 (Location), the associated trait, whether the SNP is within the coding, non-coding, or intergenic region (SNP type), and the reference article (Reference). SNPs rs3087523 and rs62155873 (^a^), rs1401209, and rs7563316 (^b^) and rs3943516 and rs6741486 (^c^) are marked as they form LD pairs with each other, respectively (see also Figure 3).

SNP ID	Ref	Alt	Risk Allele	Location	Associated Trait	SNP Type	Reference
rs4851765	C	T/A	NA	2:105396175	Severity of bronchial hyper-responsiveness	non-coding	[57]
rs114298934	C	A	NA	2:105602735	Age of menopause	non-coding	[58]
rs4851770	T	C	NA	2:105429876	Venous thrombosis	non-coding	[59]
rs186607487	A	G	A	2:105391292	Fat body mass	non-coding	[60]
rs3087523 ^a^	G	A	NA	2:105361319	BMI	coding	[61]
							[62]
					Cardiac myopathy		[63]
rs11124029	G	A	NA	2:105361304	Cardiac myopathy	coding	[63]
rs1401209 ^b^	T	G	T	2:105475760	Acute myeloid leukemia	intergenic	[19]
rs9789507	T	C	C	2:105477863			
rs7563316 ^b^	C	T	C	2:105479257			
rs17030964	C	A	C	2:105477930			
rs4851776	G	A	A	2:105484430			
rs2139109	C	T	C	2:105475835			
rs12997792	C	T	T	2:105485871			
rs13006682	T	C	C	2:105488399	PR interval	intergenic	[64]
rs17697383	C	A/T	C	2:105454164	Lung cancer in ever smokers	intergenic	[20]
rs62155873 ^a^	C	T	NA	2:105352905	Smoking status	intergenic	[62]
rs13410405	T	G	G	2:105581084	Myeloid white cell count	intergenic	[65]
rs3943516 ^c^	A	G	G	2:105577095	Platelet count	intergenic	[65]
							[66]
rs6741486 ^c^	A	G	G	2:105583173	Platelet count	intergenic	[65]
rs111945524	C	T	T	2:105482561	Unipolar depression	intergenic	[21]
rs6738207	G	A	NA	2:105373259	Height	non-coding	[62]
rs150194832	G	C	G	2:105510423	Pulse pressure	intergenic	[67]

**Table 2:** Table of CpG sites in the *FHL2* gene found in the literature. Position in chromosome based on genome version ^a^ GRCh37/hg19 or ^b^ GRCh38/hg38.

CpG ID	Position in Chromosome	Tissue	References
cg17129821	chr2:105986385 ^a^	Liver	[93]
cg19850931	chr2:105993347 ^a^	Whole blood	[43]
		Adipose tissue	[43]
cg17291435	chr2:106015527 ^a^	Liver	[93]
cg02054792	chr2:106014950 ^a^	Liver	[93]
cg10349436	chr2:106015079 ^a^	Adipose tissue	[43]
cg02445447	chr2:106015595 ^a^	Liver	[93]
cg06639320	chr2:106015740 ^a^	Whole blood	[68,78,81,83,85]
		Pancreatic islets	[68]
		Leucocytes	[94]
		Granulocytes	[92]
		Liver	[93]
		Lymphoblastoid line	[95]
		Saliva	[24]
cg22454769	chr2:106015768 ^a^	Whole blood	[68,78,81,83,85]
		Adipose tissue	[43]
		Pancreatic islets	[68]
		Leucocytes	[94]
		Granulocytes	[92]
		Liver	[93]
		Skeletal muscle	[89]
cg24079702	chr2:106015772 ^a^	Whole blood	[68,78,81,83,85]
		Pancreatic islets	[68]
		Leucocytes	[94]
		Granulocytes	[92]
		Liver	[93]
cg26344233	chr2:106015818 ^a^	Whole blood	[43]
		Adipose tissue	[43]
cg06907053	chr2:106015870 ^a^	Whole blood	[43]
		Adipose tissue	[43]
		Liver	[93]
Not specified (8 CpG sites)	chr2: 106015678–106016008 ^a^	Teeth	[88]
Not specified (12 CpG sites)	chr2:105399282–105399340 ^b^	Whole blood	[82]
Not specified	chr2:105399282 ^b^	Whole blood	[86]
Not specified	chr2:105399291 ^b^	Whole blood	[79]

## Data Availability

Not applicable.
